# An Investigation of Organic and Inorganic Mercury Exposure and Blood Pressure in a Small-Scale Gold Mining Community in Ghana

**DOI:** 10.3390/ijerph120810020

**Published:** 2015-08-21

**Authors:** Mozhgon Rajaee, Brisa N. Sánchez, Elisha P. Renne, Niladri Basu

**Affiliations:** 1 Department of Environmental Health Sciences, University of Michigan School of Public Health, 1415 Washington Heights, Ann Arbor, MI 48109, USA; E-Mail: mrajae@umich.edu; 2 Department of Biostatistics, University of Michigan School of Public Health, 1415 Washington Heights, Ann Arbor, MI 48109, USA; E-Mail: brisa@umich.edu; 3 Department of Anthropology, University of Michigan, 101 West Hall, Ann Arbor, MI 48109, USA; E-Mail: erenne@umich.edu; 4 Department of Afroamerican and African Studies, University of Michigan, 4700 Haven Hall, Ann Arbor, MI 48109, USA; 5 Faculty of Agricultural and Environmental Sciences, McGill University, 21, 111 Lakeshore Rd., Ste. Anne de Bellevue, QC H9X 3V9, Canada

**Keywords:** mercury, blood pressure, Ghana, artisanal and small-scale gold mining, ASGM

## Abstract

There is increasing concern about the cardiovascular effects of mercury (Hg) exposure, and that organic methylmercury and inorganic Hg^2+^ may affect the cardiovascular system and blood pressure differentially. In small-scale gold mining communities where inorganic, elemental Hg exposures are high, little is known about the effects of Hg on blood pressure. In 2011, we assessed the relationship between Hg exposure and blood pressure (BP) in a cross-sectional study of adults from a small-scale gold mining community, Kejetia, and subsistence farming community, Gorogo, in Ghana’s Upper East Region. Participants’ resting heart rate and BP were measured, and hair and urine samples were provided to serve as biomarkers of organic and inorganic Hg exposure, respectively. Participants included 70 miners and 26 non-miners from Kejetia and 75 non-miners from Gorogo. Total specific gravity-adjusted urinary and hair Hg was higher among Kejetia miners than Kejetia non-miners and Gorogo participants (median urinary Hg: 5.17, 1.18, and 0.154 µg/L, respectively; hair Hg: 0.945, 0.419, and 0.181 µg/g, respectively). Hypertension was prevalent in 17.7% of Kejetia and 21.3% of Gorogo participants. Urinary and hair Hg were not significantly associated with systolic or diastolic BP for Kejetia or Gorogo participants while adjusting for sex, age, and smoking status. Although our results follow trends seen in other studies, the associations were not of statistical significance. Given the unique study population and high exposures to inorganic Hg, the work contained here will help increase our understanding of the cardiovascular effects of Hg.

## 1. Introduction

Mercury is an established pollutant of global concern given the body of evidence concerning its neurological impacts [1,2]. In recent years there has been increasing concern over the impact of mercury (Hg) on the cardiovascular system, though studies have been limited with variable results. Hg, particularly organic Hg, has been associated with increases in carotid intima-media thickness and obstruction, coronary heart disease, myocardial infarctions, cerebrovascular accidents, cardiac arrhythmias, heart rate variability, atherosclerosis, carotid artery disease, and renal dysfunction [2,3]. 

The mechanisms through which Hg affects blood pressure (BP) are not well defined, but point toward oxidative stress as being key [4,5]. Increases in oxidative stress from lipid peroxidation and reduced antioxidant capacity can promote endothelial and renal dysfunction, which can increase the risk of hypertension and atherosclerosis, and result in the elevation of pulse pressure [3,6,7,8,9]. While the majority of the research shows Hg to increase oxidative stress, endothelial dysfunction, and subsequently BP, Rhee and Choi [10] found that inorganic Hg can cause a decrease in renal blood flow while renal activity remains constant, as inorganic Hg may inhibit sodium and chloride reabsorption in the kidneys [11]. 

Most research concerning the cardiovascular impact of Hg has focused on organic Hg, or methylmercury (MeHg), exposure and BP. MeHg is generally measured in hair and reflects the blood Hg concentrations at the time of hair growth (an average of one centimeter per month) [1,12]. This form of Hg exposure mainly comes from seafood consumption, and exposures have generally been associated with increases in systolic BP (SBP) and diastolic BP (DBP). For example, MeHg-associated increases in SBP have been found in studies of male whalers from the Faroe Islands [13], communities along the Brazilian Amazon [14], and adult Inuit from Nunavik, Canada [15]. Exposure-related increases in DBP have also been reported in the aforementioned study of Farose whalers as well as a study of U.S. Michigan dentists [13,16]. These results are summarized in Table 1. 

Unlike MeHg, much less is known about the cardiovascular effects of inorganic Hg exposure. Elemental Hg, a type of inorganic Hg, can be measured in urine to assess medium-term exposure, as it has a half-life of about 56–58 days in the kidneys [1,17]. Some animal studies have shown that elemental Hg exposure reduces heart rate, lowers BP, and causes arrhythmias [10,18,19]. There is some limited evidence from human population studies in support of the animal work. Epidemiologic studies have observed significant decreases in SBP with urinary Hg [16,20,21]. A study of U.S. adults with background exposure to elemental Hg observed significant decreases in SBP [20]. U.S. Michigan dentists with slightly elevated elemental Hg exposures had significant decreases in SBP with increasing urinary Hg [16]. Despite this research, there exists some contradictory evidence. A study of U.S. young adults with dental amalgams, which was ascribed to reflect long-term Hg exposure, were found to have significantly higher SBP and DBP than non-amalgam participants [21]. The study also observed a significant negative correlation of SBP and DBP with urinary Hg, a reflection of shorter-term exposure [21]. This amalgam group also had a significantly lower pulse than the non-amalgam participants, but saw a positive correlation between pulse and urinary Hg [21]. Male Slovenian Hg miners had significantly higher SBP and DBP than non-miner controls, and a positive correlation of SBP and DBP with mean past urinary Hg exposures [22]. The results of these studies are summarized in Table 1. Population surveys have indicated widespread exposure to inorganic forms of Hg in many countries (e.g., USA, Canada) [23,24], thus supporting the need for more work on the potential effects of inorganic Hg on the cardiovascular system. 

**Table 1:** Summary of Hg biomarkers (blood, hair, urine) and associations (indicated as either positive or negative) between Hg biomarkers and SBP and DBP from other studies.

Study	*n*	Mean Hg	Population	SBP	DBP	Media
Park *et al.* (2013) [20]	6607	1.03 ^a^	US NHANES adults	−	+	Blood
Valera *et al.* (2009) [15]	732	879 ^a^	Canadian Nunavik Inuit adults	+	+	Blood
Goodrich *et al.* (2012) [16]	262	0.45	US Michigan dentists (adults)	+	+	Hair
Dorea *et al.* (2005) [25]	296	3.4, 12.8 ^c^	4 Brazilian historic gold mining communities (adults)	+	+	Hair
Choi *et al.* (2009) [13]	42	7.31 ^a^	Farose whaling male adults	+	+	Hair
Fillion *et al.* (2006) [14]	251	17.8	6 Brazilian communities (adults)	+	+	Hair
Park *et al.* (2013) [20]	2201	0.51 ^a^	US NHANES adults	−	+	Urine
Goodrich *et al.* (2012) [16]	262	0.94	US Michigan dentists (adults)	−	−	Urine
Siblerud (1990) [21]	101	1.23, 3.7 ^d^	US Utahan young adults	−	−	Urine
Kobal *et al.* (2004) [22]	112	69.3 ^b^	Slovenian male Hg miners (n = 54) & control	+	+	Urine

^a^ Geometric mean; ^b^ Mean annual past urinary Hg exposure; ^c^ Dorea *et al.* (2005): 3.4 is the mean for three communities; 12.8 is the mean for one community; ^d^ Siblerud (1990): 1.23 is the mean for non-amalgam group (n = 51); 3.70 is the mean for amalgam group (n = 50).

Exposures to inorganic Hg are perhaps highest amongst members of artisanal and small-scale gold mining (ASGM) communities. This form of mining utilizes elemental Hg to bind gold, and has recently been suggested to be the largest overall contributor to global anthropogenic Hg in the atmosphere [26]. ASGM communities are unique in their potentially high exposure to elemental Hg used in the mining process [27]. Biological markers of elemental and organic Hg concentrations are predominantly measured through urine and hair, respectively [1,28,29]. 

Hypertension is a growing problem in Ghana, like other developing countries, and was the fifth largest cause of outpatient morbidity in 2008 [30]. An estimated 3.5 million Ghanaians over 15 years of age have hypertension [30]. A 1973 study reviewing rural Ghanaian villages found the prevalence of hypertension (defined as DBP > 95 mmHg) to be 2.5% for participants aged 16–54 years, and 4.5% for all participants over 16 years [31]. A 2010 review of hypertension studies in Ghana found that the prevalence of hypertension ranged from 19% to 48%; and estimated hypertension prevalence at 20% in rural areas and 25% in urban areas [30]. In addition to the problem of rising hypertension, less than one-third of hypertensive adults from Ghanaian studies in 2005 were even aware that they had hypertension [30]. 

There have been no studies to-date exploring Hg exposures in small-scale gold miners in relation to cardiovascular health. This unknown relationship with high Hg exposures and the growing problem of hypertension may pose a problem for small-scale gold miners in Ghana and across the world. This study seeks to elucidate the relationship of hair and urinary Hg with cardiovascular health through assessing BP and heart rate. It examines a unique population with elevated exposures to further determine the true impacts of inorganic Hg on BP. Based on previous research, we hypothesized that hair Hg would be associated with an increase in systolic and diastolic BP and urinary Hg would be associated with a decrease in systolic BP [13,14,15,16,20,21,22].

## 2. Materials and Methods

### 2.1. Study Populations

Two study populations were identified in the Upper East Region of Ghana: Kejetia, a small-scale gold mining community [32], and Gorogo, a subsistence farming community. Participants were recruited by household in a cross sectional study from May through July 2011. Members of each household were defined as those who eat from the same “pot”, with up to four adults interviewed per household. Institutional Review Board (IRB) approval was obtained through the University of Michigan (HUM00028444). Permission to work with the community was given by both communities’ traditional chief and the Assemblyperson representing the community in Gorogo. 

A household head was interviewed on household characteristics including demographics, water use, and cooking fuel. The household head and up to three other adult (18 years or older) household members were interviewed on their occupational and medical histories. Questions were adapted from the Ghana Demographic and Health Survey [33], and the American Thoracic Society’s Epidemiology Standardization Project [34].

In Kejetia, all households were divided into 20 clusters. In each cluster, households were randomly assigned a number, and two to three households were sampled within each cluster by drawing a number at random from a bag. Only one household from one to three clusters was interviewed per day. Due to the large geographic dispersion of the Gorogo community, households were selected through convenience sampling. A bottle was spun at a community landmark initially and the household in the bottle’s direction was selected [35]. Spinning a bottle at the preceding participating household identified subsequent households. One Gorogo participant was excluded for being a current miner. 

### 2.2. Mercury Exposure Assessment

Urine was collected to assess elemental Hg exposure and hair to assess MeHg exposure [1]. Spot urine samples (5–15 mL) were collected by participants at the time of the interview, stored at room temperature in Ghana, and frozen until analysis in the U.S. Hair was cut as close to the scalp as possible from the occipital region of the head and placed on a sticky-note and stored in a plastic bag until analysis. Only the 2 cm closest to the scalp was used for analysis [32]. 

Total Hg was measured using a Direct Mercury Analyzer-80 (DMA-80; Milestone, Inc., Shelton, CT, USA), following U.S. EPA Method 7473 [36]. Urine (500 μL) was vortexed and hair washed with acetone and deionized water before analysis. Certified reference materials (CRMs; urine: QMEQAS, Institut National de Santa Publique Quebec; hair: NIES Japan; dogfish liver: DOLT-4, National Research Council Canada) were analyzed approximately every ten samples, blanks every five samples, and sample replicates at least every nine samples. The detection limit, recovery rates of CRMs, and within-day variations for urine and hair samples are provided in Rajaee *i.e.* [37]. 

Specific gravity (SG) was measured using a pocket refractometer (PAL-10S, Atago U.S.A., Inc., Tokyo, Japan) to adjust urine samples by urinary dilution using the mean urinary specific gravity in Kejetia and Gorogo (1.016) [38,39]. The equation is as follows, where *p* refers to a participant’s personal urinary Hg and urinary SG values:

$$SG\;adjusted\;urinary\;Hg_{p}=\;\frac{\left(Avg.\;urinary\;SG-1\right)}{\left(Urinary\;SG_{p}-1\right)}\times Urinary\;Hg_{p}$$

Statistical analyses were performed with specific gravity-adjusted and non-adjusted urinary Hg.

### 2.3. Blood Pressure and Pulse Assessment

Participants had their systolic and diastolic BP and heart rate (HR), or pulse, measured three times according to American Heart Association standards (AHA) [40,41] using a manual sphygmomanometer (Omron HEM-432C; Omron Healthcare, Inc., Lake Forest, IL) about 2 cm above the right elbow, above the brachial artery as we have previously detailed [16]. Participants were asked to sit on a chair or stool (with back support when possible) for the duration of the occupational survey, to allow for participants to rest for at least 10 minutes before measurements were taken. Three measurements were averaged for each participant’s BP and pulse. Variability of each individual’s replicates averaged 4.7% (SBP), 5.4% (DBP), and 3.5% (pulse). 

BP values were classified by AHA standards for hypertensive status, with apparent hypertension defined as SBP ≥ 140 mmHg or DBP ≥ 90 mmHg [40,41]. Pulse pressure (PP) was calculated from the difference between the systolic and diastolic BP. Mean arterial pressure (MAP) was calculated from DBP + (1/3)*pulse pressure [42]. One (n = 1) male participant from Kejetia was excluded from analyses for taking anti-hypertensive medication at the time of the survey (his urinary Hg was 167.2 µg/L unadjusted and 116.3 µg/L adjusted for specific gravity). Participants with emergency high BP (SBP ≥ 180 or DBP ≥ 110 mmHg; three in Kejetia and one in Gorogo) were counseled at the time of the interview to seek advice from a medical professional.

### 2.4. BMI and Smoking

Each participant’s body mass index (BMI) was calculated by dividing the participant’s weight (kg) by his/her squared height (m), to assess obesity. Participant’s smoking history was assessed to classify participants as current, ex, and never smokers. Participants were classified as never-smokers if they had smoked less than 100 cigarettes in their lifetime. Smoking pack-years reflect the average packs (20 cigarettes per pack) of cigarettes smoked per day for the duration of a person’s smoking years.

### 2.5. Statistical Analyses

Data were analyzed using SPSS Statistical Software (v.22; IBM, Chicago, IL, USA). Urinary and hair Hg biomarkers that were not normally distributed were analyzed using Spearman correlations for bivariate analyses. Multivariable linear regressions were used to determine factors that influenced BP measures (SBP, DBP, pulse pressure, mean arterial pressure, and pulse). Sex, age, and smoking status (current and ex) were all controlled for in regression models, in addition to the exposure variables of interest: hair and urinary Hg (unadjusted and specific gravity-adjusted). Multivariable analyses were performed for the community and for miners in Kejetia. Analyses were stratified by community to better assess the impact of Hg exposures on BP, since Hg exposure was confounded by community. Models for urinary Hg were not estimated for Gorogo, as the urinary Hg distribution was very narrow (all very low values). Deviations from linear associations between Hg and BP were explored using smoothing techniques (lowess) and depicted using quintiles of the Hg distributions. Small sample sizes limited our ability to perform multilevel models such as linear mixed models despite clustering by household. However, the inferences presented here are likely conservative because the exposures of interest vary across individuals within households [43].

All linear regression models were ran including and excluding outliers with emergency high BP or SG-adjusted urinary Hg > 45 µg/L. Participants excluded for emergency high BP all had SG-adjusted urinary Hg concentrations below 6.5 µg/L. Three Kejetia participants with very high urinary Hg levels were excluded as outliers (SG-adjusted urinary Hg was 998.0, 268.4, and 106.5 µg/L for outliers). Exclusion of outliers (emergency high BP or SG-adjusted urinary Hg > 45 µg/L) improved the strength of the associations in linear regressions and are presented here. 

## 3. Results

There were 96 participants from 54 households in Kejetia and 75 participants from 26 households in Gorogo. Table 2 outlines the demographics, BMI, and smoking histories of participants, stratified by community and sex. Kejetia participants were younger than Gorogo participants, with 66.7% in Kejetia under the age of 35, but only 22.7% in Gorogo under 35 years. Kejetia participants were predominantly current miners (72.9%), but many people engaged in multiple work activities in both communities. The majority of participants in Kejetia and Gorogo had a normal BMI (18.5 to 24.9), but women in Kejetia and Gorogo had a higher prevalence of overweight BMI (25.0 to 29.9). Smoking, while less common, was almost exclusively seen in males and predominantly among miners in Kejetia. Seventeen (17.7%) of Kejetia participants and 16 (21.3%) of Gorogo participants had hypertension (SBP ≥ 140 mmHg or DBP ≥ 90 mmHg) (Table 2). 

**Table 2:** Demographics and BP of participants in Kejetia and Gorogo, and stratified by mining status.

				Kejetia			Gorogo
				All	Miners ^a^	Non-Miners ^a^	
*n* participants				96	70	26	75
*n* households				54	41	18	26
**Sex**		% Male		49 (51.0%)	42 (60.0%) ^**^	7 (26.9%)	34 (45.3%)
**Age**		Mean (SD)		31.4 (10.9) ^***^	30.6 (9.7)	33.8 (13.6)	51.5 (18.8)
**BMI ^b^**							
<18.5				5 (5.2%)	4 (5.7%)	1 (3.8%)	12 (16.0%)
18.5 to 24.9				73 (76.0%)	59 (84.3%)	14 (53.8%)	52 (69.3%)
25 to 29.9				13 (13.5%)	5 (7.1%)	8 (30.8%)	10 (13.3%)
30 or higher				5 (5.2%)	2 (2.9%)	3 (11.5%)	1 (1.3%)
Mean (SD)				22.7 (3.2)	22.1 (2.7) ^**^	24.5 (3.7)	21.8 (3.1)
**Smoking**							
Current smoker				15 (15.6%)	14 (20.0%)	1 (3.8%)	14 (18.7%)
Ex-smoker				7 (7.3%)	6 (8.6%)	1 (3.8%)	9 (12.0%)
	*n* ever-smokers with pack-years ^c^			16	15	1	14
Cigarette pack-years ^c^				15.8 (26.6)	15.1 (27.4)	25.5	3.9 (2.1)
**Blood Pressure**			Mean (SD)				
Systolic BP (mmHg)				123.5 (15.4)	122.6 (12.4)	125.8 (21.7)	126.1 (20.0)
Diastolic BP (mmHg)				76.7 (11.9)	75.2 (10.3) ^*^	80.6 (14.9)	75.4 (11.5)
Pulse Pressure ^d^ (mmHg)				46.9 (9.3) ^*^	47.5 (8.2)	45.2 (11.8)	50.7 (13.3)
Mean Arterial Pressure ^e^ (mmHg)				92.3 (12.4)	91.0 (10.4)	95.7 (16.5)	92.3 (13.4)
Pulse				77.1 (1.6) ^***^	77.0 (1.5)	77.2 (1.7)	74.6 (3.2)
**BP Classifications ^f^**							
Normal				38 (39.6%)	27 (38.6%)	11 (42.3%)	30 (40.0%)
Prehypertension				41 (42.7%)	32 (45.7%)	9 (34.6%)	29 (38.7%)
Apparent hypertension				17 (17.7%)	11 (15.7%)	6 (23.1%)	16 (21.3%)
Hypertension Stage 1				12 (12.5%)	9 (12.9%)	3 (11.5%)	8 (10.7%)
Hypertension Stage 2				5 (5.2%)	2 (2.9%)	3 (11.5%)	8 (10.7%)

**^a^** Refers to current miners and non-current miners; **^b^** For BMI, n = 74 for Gorogo; ^c^ Cigarette pack-years only include ever-smokers; ^d^ Pulse pressure = systolic − diastolic BP; ^e^ Mean arterial pressure = DBP + 0.333*pulse pressure [42]; **^f^** Normal BP is SBP < 120 and DBP < 80; prehypertension BP is SBP: 120–139 or DBP: 80–89; hypertension is SBP ≥ 140 or DBP ≥ 90; hypertension stage 1 is SBP: 140–159 or 90–99; hypertension stage 2 is SBP ≥ 160 or DBP ≥ 100; ^*^ Man-Whitney tests comparing Kejetia to Gorogo participants and Kejetia miners to non-miners: ^*^ *p* < 0.05; ^**^ *p* < 0.01; ^***^ *p* < 0.001.

Urinary and hair Hg biomarkers had skewed distributions. Descriptive statistics are reported in Table 3. In Kejetia the median (interquartile range; IQR) unadjusted urinary Hg concentration was 2.71 (1.03, 10.9) µg/L and the median SG-adjusted urinary Hg was 3.1 (1.13, 10.1) µg/L (n = 91). The median hair Hg concentration was 0.782 (0.404, 1.20) µg/g (n = 69) in Kejetia. Hg concentrations in urine (adjusted and unadjusted) and hair were significantly higher in Kejetia than Gorogo participants and Kejetia miners than non-miners. In Gorogo, urinary Hg was very low, with a median SG-adjusted Hg concentration of 0.154 (0.095, 0.261) µg/L (n = 70). Median hair Hg concentrations in Gorogo were 0.181 (0.119, 0.244) µg/g (n = 59).

**Table 3:** Mercury concentrations in urine and hair samples of participants.

		Kejetia			Gorogo
		All	Miners ^a^	Non-Miners ^a^	
**Urine**	*n*	91	67	24	70
Urinary Specific Gravity (SG)					
Mean (SD)		1.018 (0.007) ^***^	1.017 (0.007)	1.020 (0.006)	1.014 (0.006)
Urinary Hg (µg/L)					
Mean (SD)		29.4 (148.6) ^***^	37.6 (172.7) ^*^	6.61 (13.2)	0.161 (0.131)
Median		2.71	4.24	1.41	0.114
IQR ^b^		1.03, 10.9	1.24, 11.0	0.742, 5.23	0.079, 0.217
Min–Max		0.160–1372.3	0.160–1372.3	0.199–58.1	0.026–0.580
SG-adj. Urinary Hg ^c^ (µg/L)					
Mean (SD)		21.7 (107.9) ^***^	28.0 (125.3) ^***^	4.22 (6.88)	0.216 (0.194)
Median		3.10	5.17	1.18	0.154
IQR ^b^		1.13, 10.1	1.90, 12.5	0.733, 3.61	0.095, 0.261
Min–Max		0.188–998.1	0.188–998.1	0.212–25.8	0.042–1.24
**Hair Hg** (µg/g)	*n*	69	50	19	59
Mean (SD)		0.958 (0.742) ^***^	1.11 (0.807) ^**^	0.558 (0.272)	0.231 (0.202)
Median		0.782	0.945	0.419	0.181
IQR ^b^		0.404, 1.20	0.571, 1.44	0.329, 0.718	0.119, 0.244
Min–Max		0.132–3.69	0.132–3.69	0.237–1.10	0.037–1.37

**^a^** Refers to current miners and non-current miners; ^b^ Interquartile range (25th percentile, 75th percentile); ^c^ Specific gravity adjusted urinary Hg: Urinary Hg ^*^ [(1–avg. SG]/[Urine SG–1]); ^*^ Man-Whitney tests comparing Kejetia to Gorogo participants and Kejetia miners to non-miners: ^*^ *p* < 0.05; ^**^ *p* < 0.01; ^***^ *p* < 0.001.

**Figure 1:**
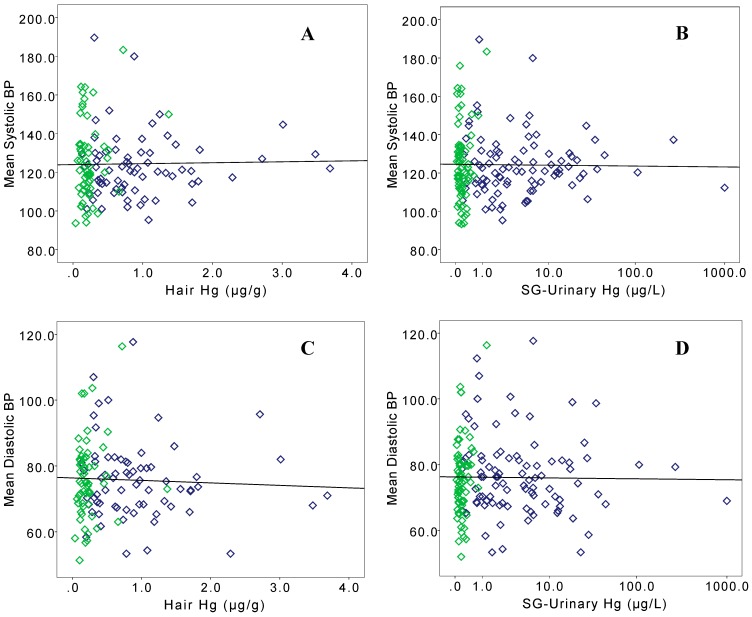
Scatterplots of BP by specific gravity-adjusted urinary Hg and hair Hg for Kejetia (dark blue) and Gorogo (green) participants. Systolic BP is represented at top with hair Hg (**A**) and SG-adjusted urinary Hg (**B**), and diastolic BP is displayed at bottom with hair Hg (**C**) and SG-adjusted urinary Hg (**D**).

**Figure 2:**
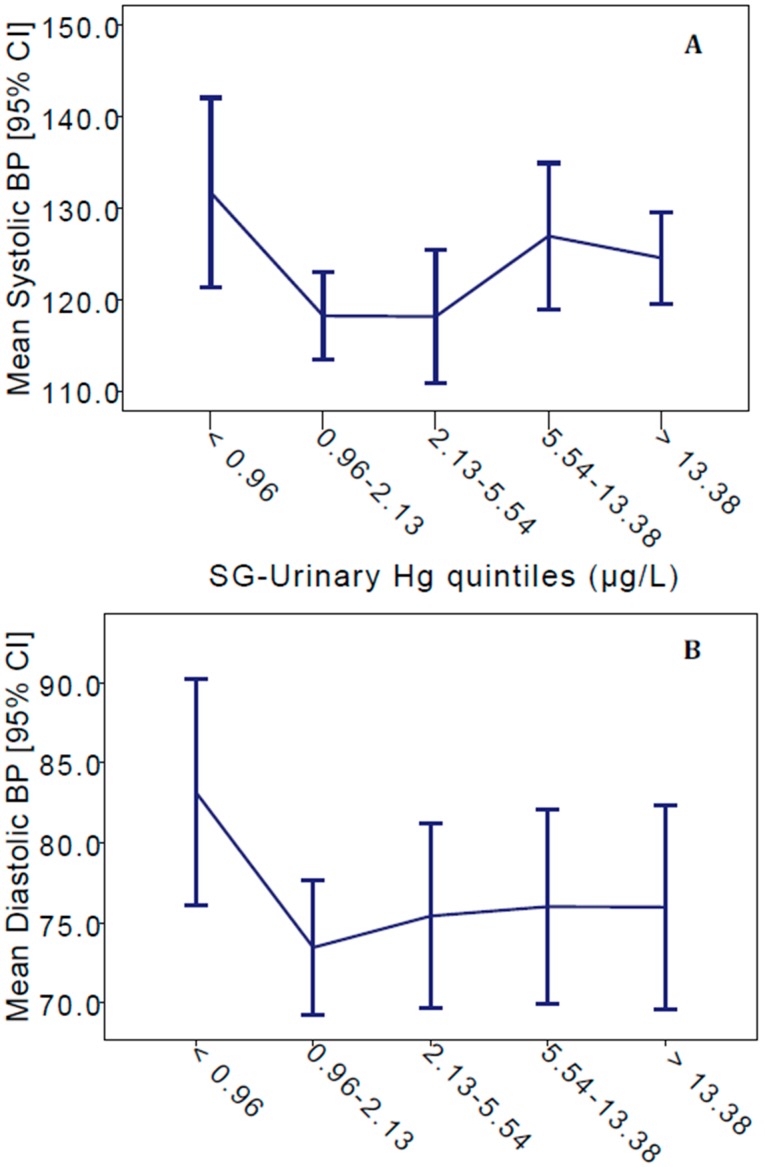
Mean systolic (**A**) and diastolic BP (**B**) by quintiles of SG-adjusted urinary Hg concentrations in Kejetia. Error bars represent 95% confidence intervals.

In bivariate analyses of Hg exposure biomarkers, SG-adjusted urinary and hair Hg were significantly correlated to each other in Kejetia (Spearman’s ρ = 0.757, *p* < 0.001) and Gorogo (ρ = 0.405, *p* = 0.002). As displayed in the scatterplots in Figure 1, there were no significant correlations between SG-adjusted urinary Hg to SBP, DBP, or MAP in Kejetia or Gorogo. Hair Hg was not significantly correlated to any BP or pulse measures in either community. PP was negatively correlated to SG-adjusted urinary Hg in Gorogo (ρ = −0.261, *p* = 0.029), but not in Kejetia. SG-adjusted urinary Hg was significantly correlated to pulse in Kejetia (ρ = −0.214, *p* = 0.042) and Gorogo (ρ = −0.323, *p* = 0.006). Figure 2 displays the mean SBP and DBP by quintiles of SG-adjusted urinary Hg in Kejetia. While the SG-urinary Hg concentrations are not significantly different by quintile group, the graph does indicate that the trend may be non-linear as elemental Hg exposures increase. 

In other bivariate analyses, age was significantly correlated to SBP (Kejetia: Pearson = 0.433, *p* < 0.001; Gorogo: Pearson = 0.346, *p* = 0.002), DBP (Kejetia: Pearson = 0.485, *p* < 0.001), PP (Gorogo: Pearson = 0.358, *p* = 0.002), and MAP (Kejetia: Pearson = 0.489, *p* < 0.001; Gorogo: Pearson = 0.276, *p* = 0.016). BMI and smoking pack-years were not significantly correlated to BP measures. Pack-years was significantly correlated to SG-adjusted urinary Hg in Kejetia (ρ = 0.220, *p* = 0.044).

Table 4 displays the results of linear regression models associating hair Hg levels and BP outcomes, while excluding outliers. Overall, the adjusted R^2^ values of the model were low (maximum was 0.350) and no significant associations were observed with hair Hg. In Kejetia and Gorogo, the association of hair Hg on SBP is positive (β = 1.45, *p* = 0.41; and β = 0.748, *p* = 0.95, respectively), and with DBP it is negative (β = −0.696, *p* = 0.65; and β = −1.71, *p* = 0.83, respectively), though none of these are statistically significant. Increasing hair Hg was associated with an increase in PP in Kejetia and Gorogo (β = 2.09, *p* = 0.15; and β = 2.46, *p* = 0.76, respectively). MAP did not have a significant association with hair Hg in Kejetia or Gorogo. The association of pulse and hair Hg was positive in Gorogo (β = 3.22, *p* = 0.13), but negative in Kejetia (β = −0.328, *p* = 0.26). The relationship with the female sex, current smoking status, and ex-smoking status were largely negative with SBP, DBP, PP, MAP, and pulse in Kejetia and Gorogo, with an exception for pulse with female sex and current smokers in Gorogo, and DBP with ex-smokers in Kejetia and Kejetia miners (all not significant). Models including participants with emergency high BP yielded similar results (data not shown). 

**Table 4:** Results of linear regression models for BP measures and hair Hg concentrations. Each line represents a separate model. Analyses excluded participants with emergency high BP statuses (n = 4). Statistically significant results are in bold font.

	Model	n	Adjusted *r*^2^	Intercept	Hair Hg (µg/g)	Female	Current Smoker	Ex-Smoker	Age (Years)
				β	β (*p*-value)				
Systolic BP	Gorogo	58	0.172	114.8	0.748 (0.95)	−13.4 (0.07)	−15.7 (0.06)	−13.8 (0.16)	**0.433 (0.001)**
	Kejetia	67	0.350	112.2	1.45 (0.41)	**−13.7 (<0.001)**	**−14.2 (0.004)**	−7.91 (0.17)	**0.600 (<0.001)**
	Kejetia: Current miners	50	0.283	109.6	1.40 (0.49)	**−11.3 (0.005)**	**−13.3 (0.017)**	−6.73 (0.30)	**0.648 (<0.001)**
Diastolic BP	Gorogo	58	−0.016	71.3	−1.71 (0.83)	−3.63 (0.44)	−3.60 (0.49)	−4.47 (0.48)	0.150 (0.07)
	Kejetia	68	0.338	60.0	−0.696 (0.65)	−4.11 (0.13)	**−9.27 (0.027)**	1.59 (0.75)	**0.636 (<0.001)**
	Kejetia: Current miners	50	0.285	60.0	−0.508 (0.75)	−3.80 (0.21)	−8.23 (0.06)	2.44 (0.62)	**0.601 (<0.001)**
Pulse pressure ^a^	Gorogo	58	0.184	43.4	2.46 (0.76)	**−9.74 (0.049)**	**−12.1 (0.029)**	−9.30 (0.16)	**0.283 (0.002)**
	Kejetia	67	0.197	50.4	2.09 (0.15)	**−9.45 (<0.001)**	−5.51 (0.16)	**−9.64 (0.040)**	0.030 (0.79)
	Kejetia: Current miners	50	0.101	49.6	1.91 (0.23)	**−7.47 (0.015)**	−5.11 (0.23)	−9.17 (0.07)	0.047 (0.72)
Mean arterial pressure ^b^	Gorogo	58	0.076	85.8	−0.890 (0.92)	−6.85 (0.19)	−7.61 (0.19)	−7.55 (0.28)	**0.245 (0.009)**
	Kejetia	67	0.316	78.6	0.057 (0.97)	**−7.44 (0.005)**	**−10.5 (0.010)**	−1.48 (0.75)	**0.580 (<0.001)**
	Kejetia: Current miners	50	0.315	76.5	0.126 (0.94)	−6.30 (0.040)	**−9.93 (0.02)**	−0.615 (0.90)	**0.617 (<0.001)**
Pulse	Gorogo	58	0.159	74.7	3.22 (0.13)	0.267 (0.83)	1.64 (0.24)	**−3.74 (0.030)**	−0.009 (0.67)
	Kejetia	67	0.066	79.4	−0.328 (0.26)	−0.874 (0.09)	−0.102 (0.90)	−1.72 (0.07)	**−0.048 (0.038)**
	Kejetia: Current miners	50	−0.015	78.6	−0.333 (0.29)	−0.571 (0.34)	−0.114 (0.89)	−1.57 (0.12)	−0.026 (0.33)

^a^ Pulse pressure is SBP **−** DBP; ^b^ Mean arterial pressure = DBP + 0.333*pulse pressure [42].

Analyses with SG-adjusted urinary Hg (Table 5) show no significant association with SBP, DBP, PP, MAP, or pulse. For Kejetia current miners, the association between SG-adjusted urinary Hg and PP was approaching significance (β = 0.205, *p* = 0.07). SBP had a positive association with SG-urinary Hg and DBP and pulse had a negative association with SG-urinary Hg, albeit non-significant. Female sex and ex-smoking status were associated with lower SBP, DBP, PP, MAP, and pulse. Current smoking status was associated with lower SBP, DBP, PP, and MAP, but higher pulse (all not significant). Models including unadjusted urinary Hg, participants with emergency high BP, or SG-adjusted urinary Hg concentrations > 45 µg/L were similar or had wider confidence intervals, with no significant associations with urinary Hg and BP outcomes (data not shown). 

**Table 5:** Results of linear regression models for BP measures and specific gravity-adjusted urinary Hg concentrations. Each line represents a separate model. Analyses excluded participants with emergency high BP statuses (n = 4) and urinary Hg concentrations < 45 µg/L (n = 3). Statistically significant results are in bold font.

	Model	n	Adjusted *r*^2^	Intercept	SG-Urinary Hg (µg/L)	Female	Current Smoker	Ex-Smoker	Age (Years)
				β	β (*p*-value)				
Systolic BP	Kejetia	86	0.216	114.6	0.010 (0.94)	**−10.6 (0.001)**	−5.28 (0.19)	**−10.0 (0.046)**	**0.466 (<0.001)**
	Kejetia: Current miners	64	0.244	108.3	0.098 (0.52)	**−9.35 (0.009)**	−5.73 (0.18)	−8.63 (0.11)	**0.622 (<0.001)**
Diastolic BP	Kejetia	86	0.279	62.1	−0.087 (0.46)	−4.31 (0.09)	−1.07 (0.75)	−3.03 (0.46)	**0.533 (<0.001)**
	Kejetia: Current miners	64	0.303	59.1	−0.106 (0.40)	−3.77 (0.19)	−0.263 (0.94)	−1.44 (0.74)	**0.595 (<0.001)**
Pulse pressure ^a^	Kejetia	85	0.109	50.6	0.116 (0.25)	**−6.85 (0.002)**	−4.77 (0.10)	**−7.30 (0.041)**	−0.006 (0.95)
	Kejetia: Current miners	64	0.112	49.2	0.205 (0.07)	**−5.57 (0.028)**	−5.47 (0.07)	−7.19 (0.06)	0.026 (0.81)
Mean arterial pressure ^b^	Kejetia	85	0.266	80.6	−0.046 (0.68)	**−6.82 (0.006)**	−2.13 (0.50)	−5.11 (0.20)	**0.473 (<0.001)**
	Kejetia: Current miners	64	0.302	75.5	−0.038 (0.76)	−5.64 (0.05)	−2.08 (0.55)	−3.83 (0.38)	**0.604 (<0.001)**
Pulse	Kejetia	85	0.055	78.6	−0.029 (0.15)	−0.766 (0.08)	0.003 (0.995)	−1.36 (0.05)	−0.028 (0.10)
	Kejetia: Current miners	64	0.005	77.9	−0.016 (0.47)	−0.254 (0.61)	0.176 (0.77)	−1.39 (0.07)	−0.019 (0.40)

^a^ Pulse pressure is SBP − DBP; ^b^ Mean arterial pressure = DBP + 0.333*pulse pressure [42].

## 4. Discussion

There is increasing concern about the cardiovascular effects of mercury (Hg) exposure, and that organic MeHg and inorganic Hg^2+^ may affect the cardiovascular system and BP differentially. In small-scale gold mining communities, inorganic, elemental Hg exposures are high for community members and miners. Little is known about the Hg-associated effects on BP in such groups, and the high exposures may represent a unique opportunity to increase understanding of Hg-associated cardiovascular risk. Here we conducted a cross-sectional epidemiological study to assess the relationships between exposures to both MeHg and inorganic Hg with BP. As with past studies from this region [32], exposures to MeHg were relatively low whereas exposures to inorganic Hg were high. When the exposure data were associated with BP measures, though relationships were in the expected direction based on past studies, they were not of statistical significance. We are limited in particular by a small number of participants with elevated urinary and hair Hg concentrations and a small sample size overall. 

In the current study, the relationship between hair Hg and SBP or DBP was not significant. The overall lack of significant finding here is perhaps due to lower hair Hg levels (*i.e*., IQR of 0.4 to 0.8 µg/g) in our study than these other studies. Fish Hg concentrations were low in the area surrounding Kejetia and across Ghana [37,44]. Fish consumption was also low in the Kejetia area of the Talensi District [32]. Omega-3 fatty acids present in fish may reduce hypertension and coronary heart disease [14,45], and this effect may be more pronounced at higher selenium and lower Hg levels [45]. The low consumption of fish, low Hg levels in fish, and potential benefits of omega-3 fatty acids in fish may all influence BP in this study. Hair samples from ASG miners or community residents may also overestimate MeHg exposure, thus clouding any association. Sherman *et al.* [46] found that hair Hg from a subsample of our Kejetia miners collected in 2011 and other Kejetia miners sampled in 2010 had a low percentage of MeHg as total Hg (7.6%–29%), suggesting that the majority of the total Hg is exogenously adsorbed elemental Hg. 

The study observed a non-significant positive association with SBP and hair Hg, which has been observed in other studies of hair of blood Hg, both usual measures of MeHg [13,14,15,16,25]. For DBP, our study found a non-significant negative association between elevated hair Hg and DBP, in contrast to other research, which have all observed an exposure-associated increase in DBP [13,14,15,16,20,25]. Goodrich *et al.* [16] estimated a 2.76 mmHg increase in DBP for every 1 µg/g increase in hair Hg. 

For exposures to inorganic Hg, the levels reported here were high as expected for current miners. The median value (4.24 µg/L) was similar to our past work with miners in this same community (3.6 µg/L) [32] as well as values found in other ASGM communities [47]. These exposure values are much greater than values from other studies in which Hg-associated decreases in SBP have been reported. For example, Park *et al.*’s [20] study of healthy U.S. adults had a 95% confidence limit of 0.47–0.54 µg/L urinary Hg and Goodrich *et al.* [16] measured urinary Hg concentrations ranging from 0.03 to 5.54 µg/L Hg (median: 0.63 µg/L). Non-miners have significantly lower urinary Hg exposures, but also significantly higher BMIs than miners in Kejetia. This could explain a higher susceptibility to hypertension if non-miners have other lifestyle factors (e.g., diet, how sedentary) in addition to BMI [48]. 

Three studies of U.S. adults observed significant decreases in SBP [16,20,21] and one observed a significant decrease in DBP with urinary Hg (Table 1) [21]. Park *et al.* [20] estimated a 0.114 mmHg decrease in SBP with a 10% increase in urinary Hg and Goodrich *et al.* [16] estimated a 1.80 mmHg decrease in SBP with every 1 µg/L increase in urinary Hg. While these studies help contextualize our study, the average urinary Hg concentrations were far lower than in our Kejetia participants. One study of Slovenian Hg miners (n = 54) with elevated historic Hg exposures observed significant increases in SBP and DBP, but had an average annual past exposure of 69.3 µg/L Hg and a current measure of 2.5 µg/L Hg in their urine [22]. 

Given this research and our lack of significant results, it is possible that inorganic Hg-associated impacts on BP may vary depending on the level of elemental Hg exposure. As observed in studies with lower urinary Hg concentrations, there may be a negative association at lower elemental Hg exposure, but a positive association at higher exposures. With only three participants with > 70 µg/L urinary Hg in Kejetia, we do not have similar exposures as observed in Kobal *et al.*’s study of Slovenian Hg miners [22], and may not be able to elucidate this relationship. The impact on BP may be more deleterious for miners with higher exposures to elemental Hg.

Hair and urinary Hg had positive associations with PP, but were not statistically significant. Other studies observed that blood Hg was positively associated with increasing PP [15,49,50]. The association of urinary Hg levels on PP has not been explored previously. Elevated PP is recognized as a risk factor for cardiovascular disease [51]. In one study, participants with higher PP had increased SBP and decreased DBP compared to participants with lower PP [9]. MAP, which is associated with the development of cardiovascular disease later in life among young men [52], is also not significantly associated with BP measures. Studies have observed decreased heart rate variability with blood Hg [49,53,54]. The negative association for pulse with urinary and hair Hg may be more pronounced in Kejetia, where Hg exposures are higher. 

While we did not see a significant relationship with hair or urinary Hg and BP, our work generally follows the trends seen in the literature. Our study was limited by a small sample size, particularly of participants with urinary Hg greater than 50 µg/L, though it should be mentioned that the average urinary Hg value in other population surveys is usually <5 µg/L Hg. Kejetia and Gorogo vary greatly in demographics, as Kejetia is a younger and more transient community. This plays an important role, as age is associated with an increase in BP, but running stratified models by community helped to account for these differences. Hypertension is rising in Ghana as it becomes more industrialized [30], underscoring the value in understanding the relationship of Hg to BP and cardiovascular health, particularly for the over 500,000 people employed in ASGM [55]. 

## 5. Conclusions

Mercury is a known toxicant that many ASGM miners are exposed to at elevated levels, but no studies have examined the relationship between Hg and BP in these populations. While we did not see a significant relationship between hair or urinary Hg and BP, our research emphasizes the need for further studies to examine a larger population with > 10 µg/L urinary Hg to help clarify the relationship of elemental Hg with BP. 
